# Long-term effectiveness and safety of lomitapide in patients with homozygous familial hypercholesterolemia: an observational case series

**DOI:** 10.1186/s13023-024-03374-9

**Published:** 2024-10-08

**Authors:** Patrizia Suppressa, Chiara Coppola, Veronica Cocco, Sallyann O’Brien

**Affiliations:** 1 Dept. of Internal Medicine and Rare Diseases Centre “C. Frugoni”, University Hospital of Bari, Piazza G. Cesare 11, Bari, 70121 Italy; 2 Chiesi Pharmaceuticals, Dublin, Ireland

**Keywords:** Homozygous familial hypercholesterolemia, Low-density lipoprotein cholesterol, Lomitapide, Lipid-lowering therapy, Apheresis, PCSK9 inhibitors

## Abstract

**Background:**

We assessed long-term real-world effectiveness and safety of lomitapide in patients with homozygous familial hypercholesterolemia (HoFH).

**Methods:**

Retrospective case series of six patients with HoFH treated with lomitapide in an Italian clinic. Changes in low-density lipoprotein cholesterol (LDL-C) during lomitapide treatment were assessed. The effect on LDL-C of PCSK9 inhibitors, apheresis and lomitapide was evaluated. Additionally, high-density lipoprotein cholesterol (HDL-C), gastrointestinal tolerability, hepatic steatosis/elasticity, transaminases, and cardiovascular events and symptoms were assessed.

**Results:**

Median age at HoFH clinical and molecular diagnoses was 25 (range 2–49) and 40 (29–71) years, respectively. Five (83.3%) had prior cardiovascular events. One patient received apheresis, which was subsequently discontinued. All patients received PCSK9 inhibitors but discontinued due to minimal effectiveness. Median (range) age at lomitapide initiation was 44 (28–73) years, with a median 47 (18–85) months’ treatment (mean dose 17.5 [5–40] mg/day). Mean (SD) baseline LDL-C was 263.2 (148.1) mg/dL, which decreased by 80% at nadir (52.8 [19.2] mg/dL) and 69% at last follow-up (81.3 [30.5] mg/dL). Four patients (66.7%) achieved LDL-C < 70 mg/dL sometime during follow-up, all of whom also achieved LDL-C < 55 mg/dL. Adverse events (AEs) were generally mild to moderate, hepatic steatosis was either absent or mild/moderate and hepatic elasticity remained normal in all but two patients (> 70 years old). All patients with reported cardiovascular symptoms had improvements in symptoms, and all patients reported stabilization or regression of intima-media thickness and atheromatous plaques.

**Conclusions:**

These long-term, real-world data demonstrate that lomitapide substantially reduced LDL-C for up to seven years. Most patients achieved LDL-C goal at some point, consistent with published Phase III trial and real-world evidence data. No patient discontinued lomitapide treatment. Further long-term follow-up in a larger patient population will be important to determine cardiovascular and other outcomes.

## Background

Homozygous familial hypercholesterolemia (HoFH) is an ultra-rare genetic disorder with an estimated prevalence of 1 in 300,000 [1–3]. HoFH is characterized by marked elevations in low-density lipoprotein cholesterol (LDL-C), resulting in an extremely increased risk of premature atherosclerotic cardiovascular disease (ASCVD) and death [1, 3–5]. Consequently, early diagnosis and intensive use of lipid-lowering therapies (LLTs) are recommended to delay or prevent ASCVD onset [3, 6]. 

HoFH is caused by bi-allelic pathogenic variants of the LDL receptor (*LDLR*) gene, or other genes affecting the LDL receptor pathway, such as *APOB*, *PCSK9* and *LDLRAP1* [3], resulting in defective (2–25% residual activity) or negative (< 2% residual activity) LDL receptor function [3, 7, 8]. There is considerable heterogeneity within the genotypic spectrum of HoFH [9], which determines the severity of the LDL-C elevation and response to LLTs [3, 7]. A genetic diagnosis of HoFH is based on the presence of the bi-allelic variants that cause familial hypercholesterolemia [6, 9]. However, genetic confirmation is hindered by several factors, including cost, availability of testing and limited sensitivity or specificity, and many patients are diagnosed too late and remain untreated or undertreated [6, 8]. 

Statins, in combination with diet and lifestyle modification, are used in the management of HoFH, and additional therapies or treatments such as ezetimibe, proprotein convertase subtilisin/kexin type 9 (PCSK9) inhibitors and lipoprotein apheresis are often required [3, 7, 8]. However, in most cases such therapeutic options do not adequately reduce the cumulative lifetime exposure of extremely high LDL-C levels specific to patients with HoFH, due to their LDLR dependence, or rapid LDL-C rebound after apheresis [3]. In a recent multinational retrospective study of patients with HoFH, only 11% achieved guideline-recommended LDL-C goals [6]. This was despite 78% receiving dual combination therapy and 42% receiving ≥ 3 LLTs, showing that even though newer, more effective therapies are now available, attainment of guideline-recommended goals remains rare [6]. 

Lomitapide is a selective inhibitor of microsomal triglyceride transfer protein (MTP) approved as an adjunctive therapy in adults with HoFH [10]. MTP is an enzyme that facilitates production of very-low-density lipoprotein (VLDL) in the liver and chylomicrons in the intestine [4, 5, 10]. By inhibiting VLDL, a precursor of LDL, lomitapide lowers LDL-C independent of the LDLR pathway [5]. Furthermore, it is the only HoFH-specific oral therapy that reduces LDL-C by inhibiting the production of apolipoprotein-B-containing lipoproteins in the liver and the small intestine, and can be titrated according to LDL-C response, tolerability and safety [10]. In a pivotal Phase III trial, lomitapide significantly reduced LDL-C over 78 weeks, enabling patients to achieve their recommended LDL-C goals, and was generally well tolerated [4]. ​ Real-world evidence of the effectiveness of lomitapide to reduce LDL-C and achieve LDL-C goals is available [5, 11, 12], but data reporting long-term effectiveness and safety in various treatment settings are still needed.

The aim of this analysis was to demonstrate current real-world evidence on the long-term effectiveness and safety of lomitapide in reducing LDL-C in patients with HoFH, and analyze LDL-C goal achievement, changes in concomitant LLT use, cardiovascular symptoms and events, and hepatic safety.

## Methods

### Study design and patients

This was an observational, retrospective case series of patients with HoFH treated with lomitapide in an Italian clinical center. Clinical information was collected from de-identified patient medical records up to February 2023.

Patients were drawn from a single center (University Hospital of Bari) and all patients with a genetic diagnosis of HoFH who had received lomitapide at any point during their treatment at the center were included in the analysis.

This analysis was approved by the Institutional Review Board of the University Hospital of Bari. The analysis was performed in accordance with the Declaration of Helsinki and written informed consent was obtained for all patients. Patients gave consent for publication of data in a retrospective manner, i.e., subsequent to data collection conducted in line with routine clinical practice.

### Data collection

Patient characteristics recorded at the time of HoFH diagnosis included age, sex, genotype and any history of CVD. Clinical information recorded at the time of lomitapide initiation included use of LLTs, lipid parameters (LDL-C, high-density lipoprotein cholesterol [HDL-C], total cholesterol and triglycerides) and hepatic parameters (alanine transaminase [ALT], aspartate transaminase [AST] and gamma glutamyl transferase [GGT]). The same parameters were recorded at each subsequent clinic visit, where available, as were details of lomitapide dosage, any adverse events (AEs) related to treatment, hepatic imaging, and any cardiovascular events or procedures.

### Objectives and assessments

The main objective was to assess the change in LDL-C levels over time, from baseline to last follow-up visit. Baseline was defined as the last clinic visit prior to the initiation of lomitapide.

Further objectives were to determine the number of patients achieving 2023 European Atherosclerosis Society (EAS)-recommended LDL-C goals for HoFH (< 70 mg/dL and < 55 mg/dL^3^) at any point during follow-up, and changes from baseline to last follow-up visit in HDL-C, ALT/AST, GGT and cardiovascular events. Liver elasticity and/or hepatic steatosis were also recorded during follow-up.

On-treatment ALT, AST and GGT were calculated as the mean of all available measurements during follow-up. Hepatic steatosis was assessed with abdominal ultrasound and liver elasticity was measured by fibroscan. Patients’ histories of cardiovascular events and procedures prior to lomitapide were recorded and any cardiovascular events, procedures or symptoms during lomitapide treatment were assessed. We also sought to assess the occurrence of AEs whilst on lomitapide treatment, including any occurrence of ALT/AST > 3x the upper limit of normal.

### Statistical analysis

Descriptive statistics were reported as mean (standard deviation [SD]) or median (interquartile range [IQR]). The percentage change from baseline in LDL-C was calculated as the difference between the patient’s baseline data prior to lomitapide treatment and at nadir or at last follow-up visit.

To assess changes in LDL-C and HDL-C level over time, the follow-up period was divided into intervals, and the mean (95% confidence interval) absolute lipid level was calculated for each interval. All measurements within any given interval were analyzed, with each patient potentially having more than one measurement within each period. As the data were more frequently collected at the start of the analysis and data collection was sparser for longer follow-ups, intervals were not equally spaced. Each category started and ended midway between the previous category and the next category (e.g., the 18-month timepoint contains all values from 15 to 21 months).

## Results

### Patients

Six patients were included in the analysis, four (66.7%) of whom were male (Table 1). Patients were given a low-fat diet to follow once initiated on lomitapide, and they also had access to a dietician/specialized dietician during treatment. Mean (SD) body mass index (BMI) was 24.2 (1.1) kg/m^2^ at lomitapide initiation, which remained normal throughout treatment despite minor fluctuations in individual patient BMI.

**Table 1:** Patient characteristics

Sex	Patient 1	Patient 2	Patient 3	Patient 4	Patient 5	Patient 6
	Male	Female	Male	Male	Male	Female
**Age**,** years**						
At HoFH clinical diagnosis	49	13	37	37	6	2
At HoFH molecular diagnosis	71	40	39	69	40	29
At lomitapide initiation (year of initiation)	73 (2018)	40 (2018)	39 (2021)	70 (2021)	48 (2015)	28 (2016)
**Lomitapide treatment duration**,** years**	3.2	4.5	1.7	1.5	7.1	6.1
**Weight**,** kg**						
At lomitapide initiation	61	65	80	74	82	67
At last follow-up	62	60	78	70	85	61
**BMI**,** kg/m**^**2**^						
At lomitapide initiation	24.4	23.9	24.2	25.6	25.0	22.2
At last follow-up	24.8	22.0	24.1	24.2	25.9	20.4
**Mutations**	*LDLR* heterozygous: c.1567G > A, p.(V523M)	*LDLR* heterozygous: c.1291G > A, p.A431T (A410T)	*LDLR* heterozygous: c.798T > A, p.Asp266Glu	*LDLR* heterozygous: c.1567G > A, p.Val523Met *LDLRAP1* heterozygous: c.711G > A p.Pro237; c.*1068C > A	*LDLR* heterozygous: c.1567G > A, p.V523M (V502M)	*LDLR* heterozygous: c.373 C > T, p.Q125X (Q104X) *PCSK9*: c.60_ 65dupGCTGCT
**Genotype**	HoFH	HoFH	HoFH	HoFH (ARH)	HoFH	HoFH
**Phenotype**	Defective/ Defective	Defective/ Defective	Defective/ Defective	Defective/ Defective	Defective/ Defective	Null/ Null
**Xanthomas at HoFH diagnosis**	Tendon	Tuberous, tendon, plane	Tendon, plane	Tendon	Tendon	Tuberous, tendon, plane
**Lipids and lipoproteins**						
**LDL-C**,** mg/dL**						
At baseline	169	325	143	122	266	554
At nadir	53	78	24	41	76	45
At last follow-up visit	67	78	120	48	124	51
**HDL-C**,** mg/dL**						
At baseline	51	77	35	47	59	28
At last follow-up visit	64	73	42	37	48	44
**Total cholesterol**,** mg/dL**						
At baseline	226	430	198	183	341	604
At last follow-up visit	137	156	174	81	182	116
**Triglycerides**,** mmol/L**						
At baseline	100	74	102	71	80	112
At last follow-up visit	31	21	59	44	65	21
**LDL-C < 70 mg/dL during follow-up**						
At any point	✓	✗	✓	✓	✗	✓
At last follow-up visit	✓	✗	✗	✓	✗	✓
**LDL-C < 55 mg/dL during follow-up**						
At any point	✓	✗	✓	✓	✗	✓
At last follow-up visit	✗	✗	✗	✓	✗	✓
**Hepatic parameters and imaging**						
**ALT**,** IU/L**						
At baseline	–	–	–	31	30	18
At last follow-up visit	53	32	48	39	–	45
**AST**,** IU/L**						
At baseline	–	–	–	30	25	25
At last follow-up visit	43	26	33	35	–	44
**GGT**,** IU/L**						
At baseline	–	–	–	46	25	10
At last follow-up visit	30	19	–	62	–	10
**Abdominal ultrasound ** [Year recorded]	Moderate steatosis [2019]	Mild steatosis [2019]	No steatosis [2022]	Mild-moderate steatosis [2022]	Moderate steatosis [2022]	Mild steatosis [2019]
**Fibroscan**,** Kpa** (Fibrosis score) [Year recorded]	4.6 (F0–1) [2019] 8.7 (F2) [2022]	6.3 (F0–1) [2019] 6.6 (F0–1) [2022]	5.5 (F0–1) [2022]	7.5 (F2) [2023]	4.0 (F0–1) [2022] 3.7 (F0–1) [2023]	4.0 (F0–1) [2019] 5.6 (F0–1) [2023]
**Cardiovascular characteristics**						
**History of major cardiovascular events and procedures at HoFH diagnosis**	CABG (1994), carotid TIA (2001), PTCA (2006), arterial thrombosis	–	Unstable angina, implantation of stents (2018 & 2019), CABG (2019)	CHF, CABG (1988 & 1997), ICD (2009), TAVI (2016)	Silent ischemia and PTCA (2001), PTCA (2002)	Implantation of stent (2010), stent restenosis (2015), CABG (2015), double aortic and mitral valve replacement
**CVD symptoms**						
At baseline	Occasional episodes of dyspnea	Asymptomatic	Frequent episodes of angina until 2019, then asymptomatic	Dyspnea due to CHF with occasional episodes of hospitalization	Asymptomatic since 2002	Asymptomatic since 2015
During lomitapide	Stable ECG	Stable ECG	No signs of ischemia on MPS	Hospitalization due to dyspnea related to pre-existing CHF, stable ECG	Stable ECG	Stable ECG
**CIMT**,** mm**						
At baseline	Right: 1.0, Left: 1.0 (2018)	Right: 1.3, Left: 1.0 (2019)	Right: 0.7, Left: 0.7 (2019)	Right: 0.7, Left: 1.2 (2020)	Right: 1.3, Left: 1.3 (2019)	Right: 1.5, Left: 1.5 (2018)
During lomitapide	Right: 1.0, Left: 1.0 (2023)	Right: 0.9, Left: 0.9 (2021)	< 0.9 (numeric values not available [2022])	Right: 0.7, Left: 1.2 (2023)	Right: 0.6, Left: 0.8 (2023)	Right: 1.5, Left: 1.5 (2022)
Change in CIMT (net response)	Stable 0.0	Regression Right: -0.4, Left: -0.1	Stable N/A	Stable 0.0	Regression Right: -0.7, Left: -0.5	Stable 0.0
**Carotid artery plaques**						
At baseline	Right: 80%, Left: 75% (2018)	No plaques (2018)	No plaques (2019)	Right: 70%, Left: 70% (2021)	Right: No plaques, Left: 20% (2019)	Right: 45%, Left: 50% (2017)
During lomitapide	Right: 65%, Left: 60% (2023)	No plaques, only fibrous irregularities of 1.2 mm (2021)	No plaques (2022)	Right: 60%, Left: 70% (2023)	Right: No plaques, Left: 20% (2023)	Right: 30–35%, Left: 35–40% (2022)
Change (net response)	Regression Right: -15%, Left: -15%	Stable N/A	Stable N/A	Regression Right: -10% Stable Left: +0.0%	Stable Right: N/A, Left: +0.0%	Regression Right: -10–15%, Left: -10–15%

Baseline was defined as the last clinic visit prior to the initiation of lomitapide. PCI includes percutaneous transluminal coronary angioplasty and implantation of stents

ALT, alanine aminotransferase; ARH, autosomal recessive hypercholesterolemia; AST, aspartate aminotransferase; BMI, body mass index; CABG, coronary artery bypass graft; CHF, chronic heart failure; CIMT, carotid intima-media thickness; CVD, cardiovascular disease; ECG, electrocardiogram; GGT, gamma glutamyl transferase; HDL-C, high-density lipoprotein cholesterol; HoFH, homozygous familial hypercholesterolemia; ICA, internal carotid artery; ICD, implantable cardioverter defibrillator; LDL-C, low-density lipoprotein cholesterol; LDLR, low-density lipoprotein receptor; MPS, myocardial perfusion scintigraphy; PCI, percutaneous coronary intervention; PTCA, percutaneous transluminal coronary angioplasty; SD, standard deviation; TAVI, transcatheter aortic valve implantation; TIA, transient ischemic attack

HoFH diagnosis was initially based on clinical presentation and/or family history in all patients, with confirmation by genetic testing at a later stage. The median age at HoFH clinical and molecular diagnoses was 25 years (range 2–49 years) and 40 years (range 29–71 years), respectively. Three patients (50.0%) were diagnosed as children and three were diagnosed following their first cardiovascular event at the ages of 37, 37 and 49 years. All patients were adults when lomitapide treatment was initiated (median age 44 years [range 28–73 years]). All patients had biallelic mutations in *LDLR* (defective/defective in five and null/null in one patient, who also had a *PCSK9* mutation) and LDL-C exceeded 400 mg/dL at diagnosis in five out of six patients with data available (missing in one patient). One patient who was defective/defective for *LDLR* was additionally compound heterozygous for *LDLRAP1* mutations, for autosomal recessive hypercholesterolemia.

A history of cardiovascular events or procedures was reported in five patients (83.3%), occurring over a period of decades in the majority (Table 1).

Values for total cholesterol and LDL-C at diagnosis were available for four patients, with means (SD) of 534.8 (69.8) mg/dL and 419.0 (37.2) mg/dL, respectively. At baseline, prior to lomitapide treatment, mean (SD) total cholesterol and LDL-C were both substantially elevated at 330.3 (150.0) mg/dL and 263.2 (148.1) mg/dL, respectively.

### Prescribed lipid-lowering therapies

All patients were receiving statin and ezetimibe therapy at baseline and throughout the duration of follow-up and all patients had received a PCSK9 inhibitor at some point (Table 2). One patient had previously received and discontinued evolocumab before the baseline timepoint and four patients (66.7%) were receiving evolocumab (*n* = 3) or alirocumab (*n* = 1) at baseline immediately prior to lomitapide treatment. The final patient received evolocumab during a temporary cessation in lomitapide therapy. Of note, no patients received down-titration of their PCSK9 dose or a washout period between PCSK9 therapy and the initiation of lomitapide treatment and there were no associated adverse effects.

**Table 2:** Lipid-lowering therapies prescribed in addition to lomitapide during follow-up

Lipid-lowering therapy	Baseline, *n* (%)	During follow-up, *n* (%)	Final visit, *n* (%)
**Statin** ^**a**^	6 (100.0)	6 (100.0)	6 (100.0)
**Ezetimibe** ^**b**^	6 (100.0)	6 (100.0)	6 (100.0)
**PCSK9 inhibitor** ^**c**^	4 (66.7)	1 (16.7)	0 (0.0)
**Apheresis**	0 (0.0)	1 (16.7)	0 (0.0)

^a^Rosuvastatin (median dose 40 mg/day [range 20–40 mg/day] and atorvastatin (median dose 40 mg/day [range 40–80 mg/day]). ^b^10 mg/day. ^c^Evolocumab 140 mg Q2W or 420 mg Q4W or alirocumab 150 mg Q2W

Baseline is defined as the clinic visit immediately prior to initiation of lomitapide treatment

PCSK9, proprotein convertase subtilisin/kexin type 9; Q2W, every two weeks; Q4W, every four weeks

The duration of PCSK9 inhibitor therapy ranged from 6 to 29 months; however, despite the dose being increased in two patients (evolocumab 140 mg every two weeks to 420 mg every four weeks) prior to discontinuation and three patients receiving the maximum dose of PCSK9 inhibitor, no patients remained on PCSK9 inhibitors at the final follow-up visit. The reason for cessation of PCSK9 inhibitors was insufficient effectiveness in all cases – even in patients with a defective/defective genotype – with a mean decrease in LDL-C of 17%, and two patients’ LDL-C levels increasing whilst on PCSK9 therapy. Other considerations for discontinuation in one patient were cost of therapy and to verify lomitapide effectiveness.

One patient received lipoprotein apheresis during follow-up, but this was discontinued at the patient’s request and when the physician determined LDL-C to be adequately controlled with lomitapide.

Patients received lomitapide for a median (IQR) of 47 (25–68) months and up to a maximum of 7 years (range 18–85 months), with a mean dose of 17.5 mg/day (range 5–40 mg/day; two patients received doses above 20 mg). All patients remained on lomitapide at the last follow-up visit. One patient’s treatment was temporarily affected by supply issues, resulting in a 20-day and a 2-week interruption, respectively. Lomitapide doses were titrated in all cases in increments of 5 or 10 mg as required, to minimize adverse events and find the lowest effective dose.

### The effect of lomitapide on LDL-C

**Fig. 1:**
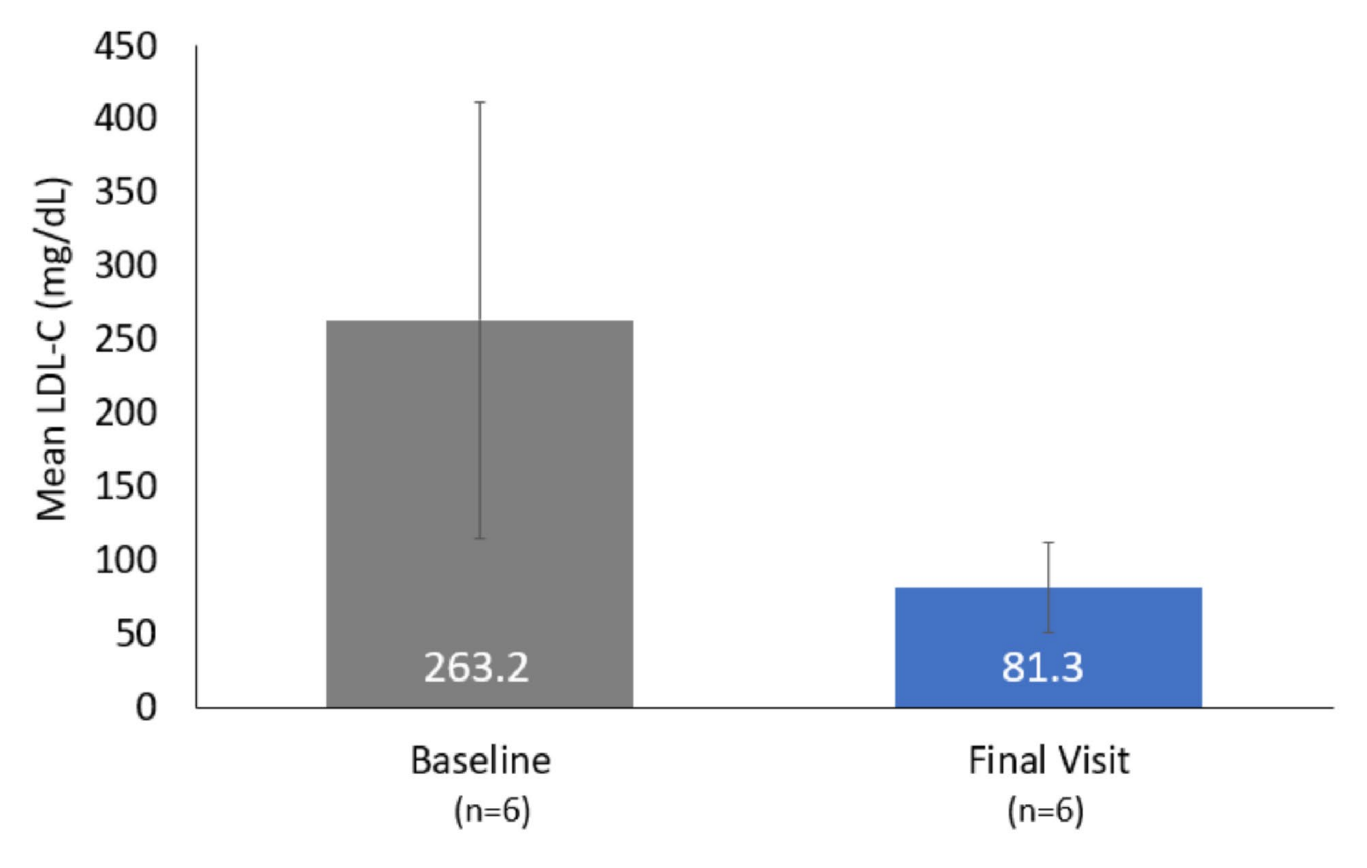
Comparison of LDL-C levels at baseline and final visit Error bars represent standard deviation LDL-C, low-density lipoprotein cholesterol

**Fig. 2:**
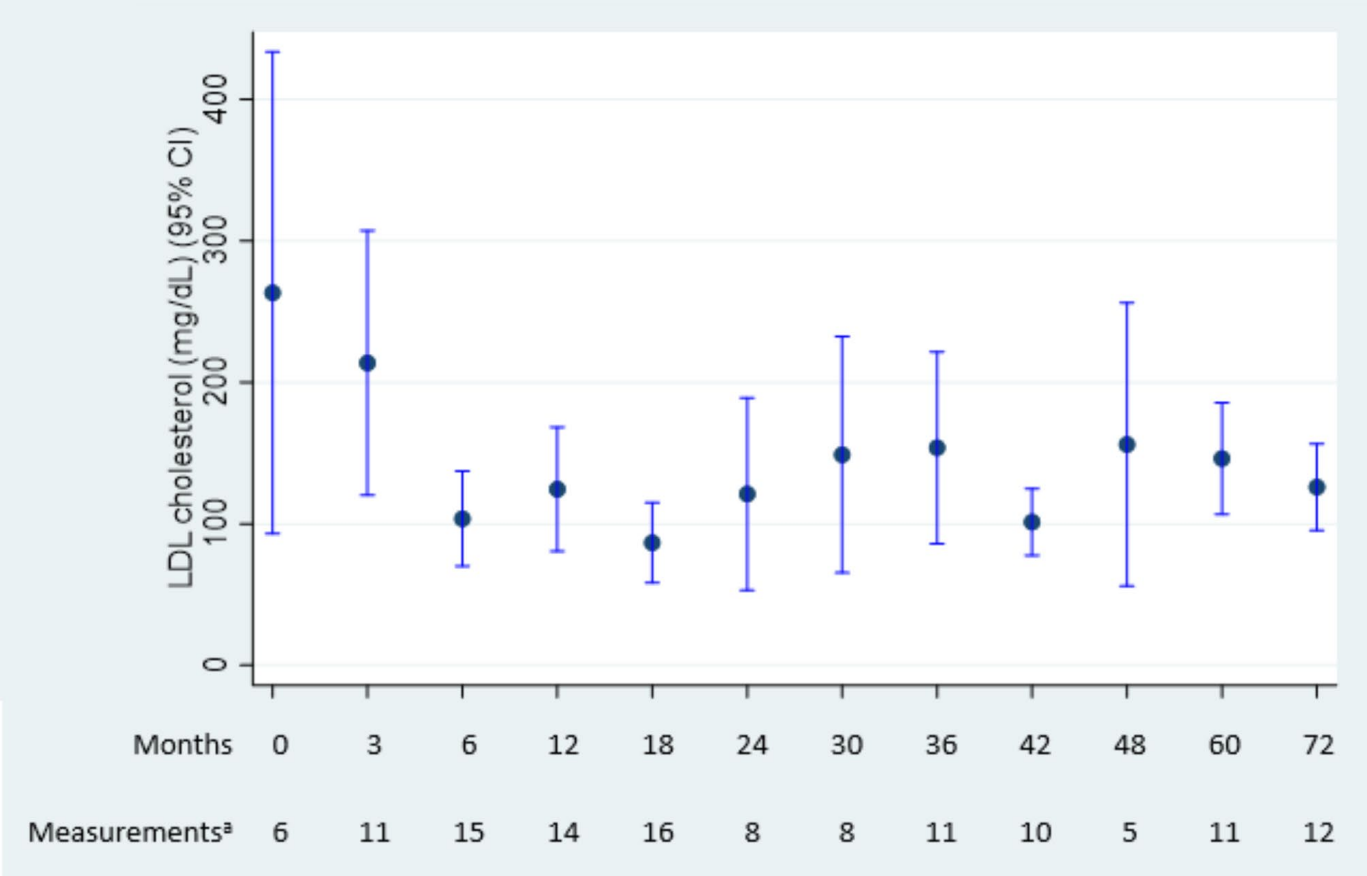
Changes in LDL-C during the follow-up period Mean LDL-C in each period is plotted along with a corresponding 95% CI ^a^The number of measurements analyzed within the given intervals is shown at the bottom of the graph. Each patient could have more than one measurement within each period. As the data were more frequently collected at the start of the analysis and data collection was sparser for longer follow-ups, intervals were not equally spaced. Each category started and ended midway between the previous category and the next category CI, confidence intervals; LDL-C, low-density lipoprotein cholesterol

LDL-C decreased by 69% during lomitapide treatment from a mean (SD) of 263.2 (148.1) mg/dL at baseline to 81.3 (30.5) mg/dL at last follow-up (Fig. 1). LDL-C levels were rapidly and substantially reduced upon lomitapide initiation (Fig. 2). The mean (SD) nadir LDL-C was 52.8 (19.2) mg/dL, representing an 80% reduction from baseline.

All patients achieved LDL-C < 100 mg/dL at some point during follow-up and four patients (66.7%) were at this goal at the end of follow-up. Four patients (66.7%) achieved LDL-C < 70 mg/dL at some point during follow-up, all of whom also achieved LDL-C < 55 mg/dL. At the last follow-up visit, three patients (50.0%) had LDL-C < 70 mg/dL, two of whom (33.3%) had LDL-C < 55 mg/dL (Table 1). The patient whose LDL-C was < 70 mg/dL but > 55 mg/dL was receiving 10 mg/day at last follow-up, whereas the patients with LDL-C < 55 mg/dL were receiving daily doses of 10 mg and 40 mg, respectively.

As expected, there was no effect of *LDLR* genotype on LDL-C reduction; all patients had substantial reductions in LDL-C from baseline to last follow-up visit, including those patients with additional *PCSK9* and *LDLRAP1* mutations.

### The effect of lomitapide on HDL-C

**Fig. 3:**
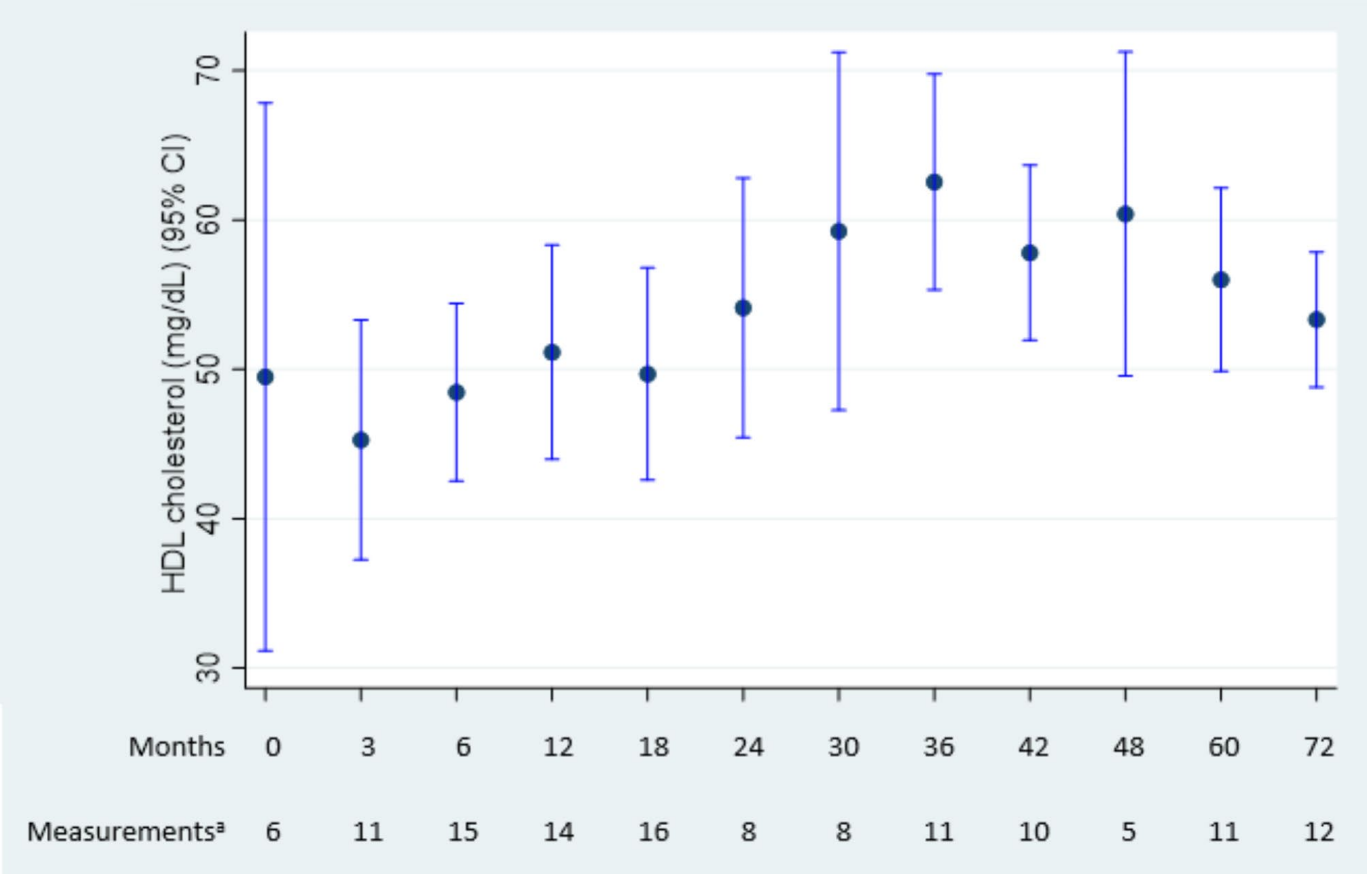
Changes in HDL-C during the follow-up period Mean HDL-C in each period is plotted along with a corresponding 95% CI ^a^The number of measurements analyzed within the given intervals is shown at the bottom of the graph. Each patient could have more than one measurement within each period. As the data were more frequently collected at the start of the analysis and data collection was sparser for longer follow-ups, intervals were not equally spaced. Each category started and ended midway between the previous category and the next category CI, confidence intervals; HDL-C, high-density lipoprotein cholesterol

Mean (SD) HDL-C was similar at baseline and last follow-up visit: 49.5 (16.0) and 51.3 (12.8) mg/dL, respectively. Mean absolute HDL-C values over the follow-up period are shown in Fig. 3. Despite some minor fluctuations, HDL-C levels remained relatively stable or increased throughout the follow-up period.

### Hepatic outcomes

There were mild, clinically insignificant increases in ALT and AST < 3x upper limit of normal (ULN) from baseline to last follow-up visit. Mean (SD) ALT was 26.3 (5.9) IU/L at baseline and 43.4 (7.3) IU/L at last follow-up and mean (SD) AST was 26.7 (2.4) IU/L at baseline and 36.2 (6.7) IU/L at last follow-up. Only one patient had a temporary increase in transaminases > 3x ULN, which was managed by a 5 mg dose reduction, and there were no elevations > 5x ULN. There was minimal change in GGT from baseline to last follow-up visit (mean [SD]: 27.0 [14.8] and 30.3 [19.7] IU/L, respectively). Median (IQR) on-treatment ALT, AST and GGT values were 48.0 (37.0–74.0), 41.0 (33.0–53.0) and 22.0 (13.0–31.3) IU/L, respectively.

Abdominal ultrasound and hepatic elastography/fibroscan results available during lomitapide treatment are shown in Table 1. There were no hepatic imaging results available at baseline or pre-lomitapide treatment. Abdominal ultrasound results during treatment follow-up showed no hepatic steatosis in one patient and mild to moderate steatosis in the remaining patients. Hepatic elastography showed that liver elasticity measurements were normal in four patients (range 3.7–6.6 kPa), with two patients (71 and 77 years old) having moderate increases in hepatic stiffness at the last follow-up visit (7.5 KPa [F2] and 8.7 Kpa [F2], respectively). The latter patient’s prior measurement three years previously was normal (4.6 Kpa).

### Cardiovascular outcomes

Despite the majority of patients having a history of cardiovascular events or procedures prior to lomitapide initiation, CVD symptoms were relatively stable at lomitapide initiation, with three patients being asymptotic. This was likely due to patients receiving standard-of-care LLTs and other cardiovascular standard-of-care drugs at baseline.

Overall, the data was such that no causal relationship between lomitapide treatment and cardiovascular outcomes could be determined. The only cardiovascular event recorded during lomitapide treatment was one patient being hospitalized for coronary angiography related to pre-existing heart failure (NYHA III–IV). This patient had previously been hospitalized for heart failure in the year prior to lomitapide initiation whilst receiving alirocumab. All patients with CVD symptoms prior to lomitapide initiation were observed to have improvements in their symptoms, particularly angina (Table 1). One patient showed no signs of ischemia on myocardial perfusion scintigraphy and the other symptoms were otherwise stable during lomitapide treatment. Patients also had stable echocardiogram traces during lomitapide treatment follow-up.

Carotid intima-media thickness (CIMT) and carotid plaques remained stable, with no progression during lomitapide treatment follow-up, although one patient who had received lomitapide for 4.5 years had fibrous irregularities of 1.2 mm at last follow-up. Two patients had CIMT regression: one patient, who initiated lomitapide therapy in 2015, had CIMT regression from 2019 to 2023 (Right carotid artery: from 1.3 to 0.6 mm [-54%]; left carotid artery: from 1.3 to 0.8 mm [-38%]. The other patient, who initiated lomitapide therapy in 2018, had CIMT regression from 2019 to 2021 (Right carotid artery: from 1.3 to 0.9 mm [-31%]; left carotid artery: from 1.0 to 0.9 mm [-10%]. Two patients also had regression of atheromatic plaques of the carotid arteries. In the first patient, stenosis of the right and left carotid arteries upon lomitapide initiation was 45% and 50%, respectively; after 6 years of treatment, the rate of stenosis was 30–35% on the right and 35–40% on the left. In the second patient, stenosis at baseline was 80% in the right carotid artery and 75% in the left carotid artery; after 5 years of treatment, the rate of stenosis was 65% on the right and 60% on the left.

### Adverse events

Overall, the available tolerability data suggested that lomitapide treatment was generally well tolerated and adverse events were mild to moderate. One patient had raised ALT/AST > 3x ULN, in whom the dose of lomitapide was reduced from 15 to 10 mg and a stabilization in liver enzymes was subsequently observed. Three patients (50.0%) reported diarrhea; two whilst receiving doses of 20 mg, which was treated in both cases with temporary dose reductions to 15 mg and also resulted in temporary treatment interruption for one of the patients who was poorly compliant to a low-fat diet. One patient reported diarrhea upon initiation of lomitapide treatment at a dose of 5 mg, which was treated with loperamide. One patient reported a single case of pruritis, which was not considered treatment related.

## Discussion

This long-term, real-world, retrospective case series of HoFH treatment demonstrates that lomitapide substantially reduced LDL-C and helped patients achieve LDL-C goals in a series of patients with a history of CVD as well as late diagnosis and treatment. These patients are reflective of other published HoFH cohorts [4, 5, 11–14], with five of six patients having CVD and a mean baseline LDL-C of 263.2 mg/dL on standard LLTs before treatment with lomitapide. Lomitapide doses were titrated according to clinical response in all patients, and there was no influence of *LDLR* genotype on LDL-C reduction associated with lomitapide, with similar reductions in both null/null and receptor defective patients, as expected. HDL-C levels remained stable or increased slightly throughout lomitapide treatment. Hepatic steatosis was either absent or mild to moderate after up to 7 years treatment with lomitapide. Hepatic elasticity remained normal in all but two older patients (> 70 years old) who had moderate hepatic stiffness at the last follow-up visit; however, one of the patients had no hepatic imaging data at baseline and therefore the hepatic stiffness before lomitapide treatment is unknown. Only one patient had a temporary increase in transaminases > 3x ULN, which was managed with a temporary dose reduction. The few adverse events reported were mild and manageable. Finally, improvements in CVD symptoms were observed in all patients with reported cardiovascular symptoms pre-lomitapide treatment, and there was stabilization or regression of CIMT and atheromatous plaques in all patients.

These effectiveness, tolerability and safety findings are in line with previous studies of lomitapide in patients with HoFH [4, 5, 11–14] and support that marked reductions in LDL-C can be achieved and gastrointestinal (GI) tolerability and long-term hepatic safety can be well managed in the real-world clinical setting. Long-term follow-up is usually not feasible in clinical trials, and the rarity of HoFH can make a powered clinical trial difficult to conduct. Therefore, real-world evidence such as this case series should be considered complementary to clinical trial data, with longer follow-up and the benefit of better reflecting the variability of patients, as seen in-clinic.

Although a causal relationship between lomitapide treatment and cardiovascular outcomes could not be determined in the present case series, it is of clinical interest to note the positive findings that there appeared to be no progression of atherosclerosis, with some signs of CIMT regression, which is the ultimate goal of aggressive lipid lowering in HoFH. It remains to be determined if these results represent a meaningful or sustainable outcome for future CVD events.

Due to the rarity of HoFH, it is difficult to analyze cardiovascular outcomes related to lomitapide treatment, but improvements in CVD symptoms and atherosclerotic burden has been observed in previous lomitapide studies [5, 11, 15, 16]. One retrospective real-world study found there were fewer cardiovascular events in patients treated with lomitapide compared with a cohort treated with lipoprotein apheresis [11], and another multinational real-world cohort study demonstrated a MACE incident rate of 7.4/1000 person-years in the two years after lomitapide treatment versus 21.2/1000 person-years in the two years prior, although this difference was not statistically significant [5]. 

In this analysis, lomitapide doses were up-titrated in all patients in 5 or 10 mg increments. The increments of 5 mg were used initially to reduce adverse events – particularly GI events – and find the lowest effective lomitapide dose to achieve LDL-C goals. Once the patients had demonstrated a good tolerance to the drug, the dose increments were increased to 10 mg, if necessary, to reach LDL-C goal. Four patients in this analysis were treated with a maximum dose of 20 mg or less, one patient received a maximum of 25 mg and one patient was able to increase their dose to 40 mg at last follow-up visit. With this flexible approach it was possible to minimize adverse events while aiming to treat to LDL-C goal. LDL-C was at, or close to, LDL-C goal at last follow-up for all patients, demonstrating that appropriate titration of lomitapide is important to enable patients to achieve their LDL-C goal, and that greater reductions in LDL-C are generally seen with more intensive lipid-lowering therapy. It should be noted that during the treatment of these patients, the LDL-C goals were < 100 mg/dL in patients without ASCVD (applicable to one patient in our analysis), or < 70 mg/dL in patients with ASCVD (applicable to the other five patients in our analysis), and therefore these were the clinical goals of the treating physicians [17]. In 2023, the updated EAS consensus statement recommended an LDL-C goal of < 70 mg/dL for adults with HoFH, or if patients have established ASCVD, or ASCVD risk factors such as elevated Lp(a) or diabetes mellitus, the recommended goal is < 55 mg/dL.^3^ However, despite combination therapy with LLTs and newer therapies available such as PCSK9 inhibitors, attainment of guideline-recommended goals remains rare [6]. 

The administration of lomitapide also resulted in a reduced necessity for other adjunctive LLTs. In the one case in this analysis where lipoprotein apheresis was administered, it was later discontinued at the patient’s request and in agreement with the physician, when LDL-C levels were substantially reduced. This is similar to findings from other studies with lomitapide, in which patients have either permanently discontinued or reduced the frequency of apheresis treatments whilst on lomitapide therapy [4, 5, 12]. Reasons for the other patients in this analysis not receiving apheresis included advanced age and comorbidity considerations, with lomitapide being preferred instead by both patient and physician. Apheresis can be time-consuming, uncomfortable and is associated with a reduced quality of life, including an increased risk of depression [18, 19]. Attending regular apheresis sessions also places a significant burden on patients in terms of travel time – which may also come with an associated financial burden – as well as interruption to normal activities and social life, and a potential impact on education and employment prospects [18–20]. The reduced use of apheresis in this case series is reflective of recent real-world data in HoFH showing 13–50% of patients are receiving apheresis, which is a smaller percentage than seen in the past [5, 11, 21]. 

In contrast to the limited use of apheresis, all patients in this analysis received PCSK9 inhibitors at some point; mostly at baseline, prior to lomitapide treatment. However, no patients remained on PCSK9 inhibitors at the final follow-up visit, with the reason for cessation being lack of effectiveness in all cases. This was despite some patients receiving PCSK9 inhibitor therapy for many months, and/or the dose being increased. In contrast, LDL-C levels were rapidly and substantially reduced upon initiation of lomitapide. The lack of effectiveness seen with PCSK9 inhibitors was not totally unexpected, as they rely on residual LDL receptor function [22] and have limited or no effect in patients with null mutations or mutations in *LDLRAP1.* [7, 23] However, some degree of response might be expected in five of the six patients with defective mutations [24, 25], so the fact that these therapies were also discontinued due to lack of effectiveness in defective/defective *LDLR* genotype patients is a notable finding. This, along with the greater convenience of oral therapies over injectable therapies and the high cost of PCSK9 inhibitors, also raises the question of the usefulness of prescribing these therapies in null/null HoFH patients. The 2023 EAS guidance recommends a short trial period in receptor defective patients, before continuing LDLR independent therapies [3]. Lomitapide lowers LDL-C independently of the LDLR pathway [5] and, consistent with previous studies [4, 5, 12, 26], LDL-C reduction was independent of the LDLR mutation status.

Lomitapide was generally well tolerated in this analysis and the main adverse events reported were transient increases in transaminases < 3x ULN in all but one case, or mild to moderate GI symptoms that were manageable with modifications in dosage or temporary dose reduction. GI tolerability was achieved with a strong emphasis by the treating physician on the importance of adhering to a low-fat diet, and patients had access to a specialised dietician, especially in the last year of treatment; this was also reflected by the mean BMI remaining stable during treatment. Lomitapide did not reduce plasma HDL-C levels and hepatic elasticity remained normal in the majority of patients.

The real-world data collected in this retrospective analysis emphasize the need for intensive use of lipid-lowering therapy to reach LDL-C goal, and also highlights the benefits of a tailored approach to treatment. Interestingly, at last follow-up, all patients were treated only with statins, ezetimibe and lomitapide, despite also having access to apheresis and PCSK9 inhibitors. Our results suggest that, in many patients with HoFH, lomitapide could be considered as the preferred adjunct to statins and ezetimibe.

Of note, half of the patients included in this analysis were not diagnosed with HoFH until well into adulthood, and it required a major cardiovascular event or procedure before the diagnosis of HoFH was made. Furthermore, some patients had not been treated at specialist centers before their first cardiovascular event, despite a family history indicative of genetically raised LDL-C and/or characteristic symptoms such as xanthomas. This highlights the need for better education around this condition to facilitate earlier diagnosis, referral to a specialist lipid clinic and prompt treatment with maximally tolerated lipid-lowering therapies.

A strength of this analysis is the long-term follow-up and detailed information collected, with patients receiving lomitapide for up to 7 years treatment. Long-term safety and effectiveness data are important, as HoFH patients require life-long intensive lipid-lowering treatments to adequately reduce LDL-C to goal levels and prevent CVD development or further progression. Limitations of this analysis included the small sample size, due to the rarity of HoFH, and the retrospective, observational design. Furthermore, as the patients were on a range of background LLTs during lomitapide treatment, the clinical effect of these therapies cannot be discounted. There was also no formal measure of adherence to lomitapide, other LLTs, or to a low-fat diet, and some patients experienced interruptions to treatment because of medication supply issues and adverse events, which could have had a bearing on the results. Finally, since this was a single-center case series, this may not be reflective of experiences in other clinical centers. Additional long-term studies with a larger patient population are required to further evaluate the cardiovascular and other outcomes in lomitapide treated patients.

## Conclusions

In conclusion, in this real-world analysis, long-term treatment with lomitapide effectively reduced LDL-C levels in patients with HoFH, enabling the majority of patients to achieve their LDL-C goals. This is consistent with phase III and other published real-world evidence. The available data indicated that lomitapide was generally well tolerated with a low-fat diet, and adverse events were mild to moderate and manageable with temporary dose adjustment. Hepatic steatosis was either absent or mild to moderate and hepatic elasticity remained normal in all but two patients > 70 years old. Most patients chose not to have lipoprotein apheresis and PCSK9 inhibitors were ineffective and discontinued in all cases, demonstrating that LDL-C goal reductions can be achieved in most cases with lomitapide in addition to statins and ezetimibe. This case series provides the basis for additional real-world evidence to be generated, and to further assess the long-term effectiveness, safety and cardiovascular outcomes of lomitapide in HoFH.

## Data Availability

The datasets generated and/or analyzed during the current case series are not publicly available due to reasons of sensitivity but are available from the corresponding author on reasonable request.
